# [Rb(18-crown-6)][Rb([2.2.2]-cryptand)]Rb_2_Sn_9_·5NH_3_

**DOI:** 10.1107/S1600536811013997

**Published:** 2011-04-22

**Authors:** Stefanie Gaertner, Nikolaus Korber

**Affiliations:** a Institut für Anorganische Chemie, Universität Regensburg, Universitätsstrasse 31, 93053 Regensburg, Germany

## Abstract

The crystal structure of the title compound, poly[[(4,7,13,16,21,24-hexa­oxa-1,10-diaza­bicyclo­[8.8.8]hexa­cosa­ne)rubidium] [[(1,4,7,10,13,16-hexa­oxacyclo­octa­deca­ne)rubidium]di-μ-rubidium-μ-nona­stannide] penta­ammonia], {[Rb(C_18_H_36_N_2_O_6_)][Rb_3_Sn_9_(C_12_H_24_O_6_)C_12_H_24_O_6_)]·5NH_3_}_*n*_ represents the first ammoniate of a Zintl anion together with two different chelating substances, namely 18-crown-6 and [2.2.2]-cryptand. The involvement of these large mol­ecules in the crystal structure of [Rb(18-crown-6)][Rb([2.2.2]-cryptand)]Rb_2_Sn_9_·5NH_3_ leads to the formation of a new structural motif, namely one-dimensionally extended double strands running parallel to [100] and built by Sn_9_^4−^ cages and Rb^+^ cations. The double strands are shielded by 18-crown-6 and [2.2.2]-cryptand. The cations are additionally coordin­ated by ammonia mol­ecules. One of the four independent Rb^+^ cations is disordered over two sets of sites in a 0.74 (2):0.26 (2) ratio.

## Related literature

For a recent review on nine-atom group 14 clusters in solution, see: Scharfe & Fässler (2010 ▶). For Zintl clusters in the solid state see: Fässler (2001 ▶); Hoch *et al.* (2003 ▶). The coordination of two cations by 18-crown-6 usually results in two-dimensional layers (Hauptmann & Fässler, 2002 ▶, 2003*a* ▶,*b* ▶), when three cations are coordinated by the latter, one-dimensional single strands are observed (Fässler & Hoffmann, 1999 ▶). The use of less [2.2.2]-cryptand gives two-dimensional double layers (Hauptmann *et al.*, 2001 ▶), whereas larger amounts result in one-dimensional single strands (Burns & Corbett, 1985 ▶) or isolated nona­stannide clusters without direct cation contacts (Corbett & Edwards, 1977 ▶).
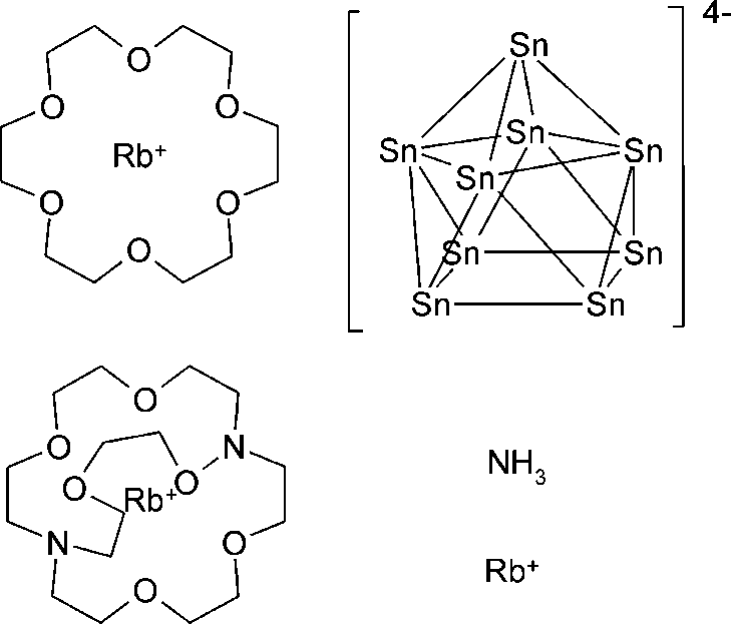


## Experimental

### 

#### Crystal data

[Rb(C_12_H_24_O_6_)][Rb_3_Sn_9_(C_18_H_36_N_2_O_6_)]·5NH_3_
*M* *_r_* = 2136.04
Monoclinic, 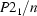

*a* = 10.790 (2) Å
*b* = 15.600 (3) Å
*c* = 36.960 (7) Å
β = 91.20 (3)°
*V* = 6220 (2) Å^3^
*Z* = 4
Mo *K*α radiation
μ = 6.75 mm^−1^
*T* = 123 K
0.25 × 0.2 × 0.15 mm

#### Data collection

Stoe IPDS diffractometer
Absorption correction: analytical (*X-SHAPE*; Stoe & Cie, 2002 ▶) *T*_min_ = 0.057, *T*_max_ = 0.119
36425 measured reflections
11435 independent reflections
7473 reflections with *I* > 2σ(*I*)
*R*_int_ = 0.099

#### Refinement

*R*[*F* ^2^ > 2σ(*F* ^2^)] = 0.043
*wR*(*F* ^2^) = 0.112
*S* = 0.84
11435 reflections
608 parameters
H-atom parameters constrained
Δρ_max_ = 2.77 e Å^−3^
Δρ_min_ = −1.18 e Å^−3^

### 

Data collection: *X-AREA* (Stoe & Cie, 2002 ▶); cell refinement: *X-AREA*; data reduction: *X-RED* (Stoe & Cie, 2002 ▶); program(s) used to solve structure: *SHELXS97* (Sheldrick, 2008 ▶); program(s) used to refine structure: *SHELXL97* (Sheldrick, 2008 ▶); molecular graphics: *DIAMOND* (Brandenburg, 2001 ▶); software used to prepare material for publication: *publCIF* (Westrip, 2010 ▶).

## Supplementary Material

Crystal structure: contains datablocks I, global. DOI: 10.1107/S1600536811013997/hp2005sup1.cif

Structure factors: contains datablocks I. DOI: 10.1107/S1600536811013997/hp2005Isup2.hkl

Additional supplementary materials:  crystallographic information; 3D view; checkCIF report

## Figures and Tables

**Fig. 1.**
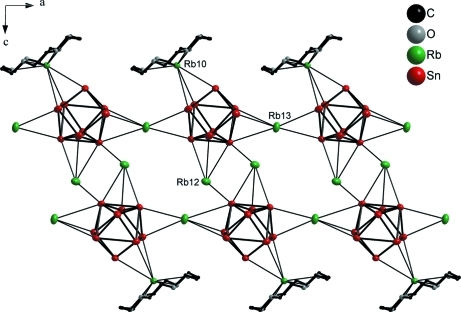
: One-dimensionally extended double strands along the crystallographic a axis built by Rb12 and Rb13 (split position is not shown), 18-crown-6 molecules around Rb10 shield the strands. H atoms of 18-crown-6 are omitted; ellipsoids of all non-hydrogen atoms are given with a probability factor of 50%.

**Fig. 2.**
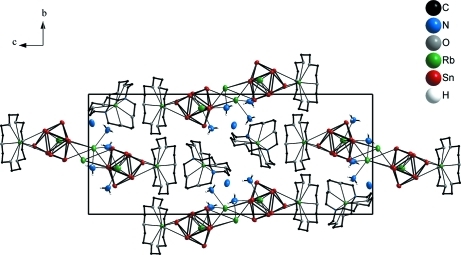
: The unit-cell content of [Rb(18-crown-6)][Rb([2.2.2]-cryptand)]Rb2Sn9 × 5NH3 shows the separation of the strands by [2.2.2]-cryptand molecules around Rb11; the coordination spheres of the Rb cations are saturated by ammonia molecules. H atoms of 18-crown-6 and [2.2.2]-cryptand are omitted; ellipsoids of all non-hydrogen atoms are given with a probability factor of 50%.

## supplementary crystallographic information

### Comment

Compounds which contain elements of group 14 elements in negative oxidation states can be obtained by reacting an electropositive metal with the favoured element in solid state reactions at high temperatures. Some of these Zintl phases contain molecular homoatomic clusters preformed in solid state (Hoch *et al.*, 2003, Fassler, 2001). The dissolution of these cluster containing Zintl compounds in an appropriate solvent is a common route to obtain homoatomic building blocks in solution (Scharfe & Fassler, 2010). Dissolving the binary material Rb_12_Sn_17_, which contains four and nine atom clusters of tin, in liquid ammonia in presence of 18-crown-6 and [2.2.2]-cryptand yields a dark red solution, the colour of which is characteristic for polystannides in solution. After storage at 236 K for five months dark red, rod shaped crystals could be isolated, which only include the nine atom clusters Sn_9_^4-^ and four Rb^+^, of which one Rb cation is coordinated by 18-crown-6 and one Rb^+^ is situated in the cavity of a [2.2.2]-cryptand molecule. The coordiantion of two cations by 18-crown-6 usually results in two-dimensional layers (Hauptmann & Fassler, 2003*a*,*b*; Hauptmann & Fassler, 2002), when three cations are coordinated by the latter, one-dimensional single strands are observed (Fassler & Hoffmann, 1999). The use of less [2.2.2]-cryptand gives two-dimensional double layers (Hauptmann *et al.*, 2001), whereas larger amounts result in one-dimensional single strands (Burns & Corbett, 1985) or isolated nonastannide clusters without direct cation contacts (Corbett & Edwards, 1977). The presence of the two different chelating compounds offers the possibility of a new structral motif in nonastannide compounds. Here, one-dimensional double strands of nonastannide cages linked by Rb^+^ (Rb10, Rb12, Rb13) along the crystallographic *a* axis are observed. These double strands are shielded by 18-crown-6 molecules, which surround Rb10 (Fig.1). [2.2.2]-cryptand molecules around Rb11 isolate the double strands from each other. The coordination spheres of the Rb cations are saturated by ammonia molecules, additionally one ammonia molecule of crystallization is found (Fig. 2).

### Experimental

0.36 g Rb_12_Sn_17_ (0.115 mmol), 0.06 g 18-crown-6 (0.25 mmol) and 0.09 g [2.2.2]-cryptand (0.25 mmol) were placed under Argon atmosphere in a baked out reaction vessel. Afterwards, 15 ml of dry liquid ammonia were condensed onto the reaction mixture, which yielded a dark red solution. After storage for five months at 236 K, dark red crystals could be obtained.

### Refinement

For one Rubidium atom a split model was applied due to large anisotropic displacement, yielding refined sof values of 0.874 (Rb13) respectively 0.126 (Rb14). The H atoms of four ammonia molecules were constructed at geometrically reasonable positions using a riding model (HFIX). For one ammonia molecule, which does not show contacts to cations, it did not seem reasonable to construct H atoms. Due to the extreme sensitivity towards moisture the implacement of a water molecule can be excluded. The H atoms of 18-crown-6 and [2.2.2]-cryptand were also located by using the HFIX instructions.

### Crystal data

**Table:**

[Rb(C_12_H_24_O_6_)][Rb_3_Sn_9_(C_18_H_36_N_2_O_6_)]·5NH_3_	*F*(000) = 3980
*M**_r_* = 2136.04	*D*_x_ = 2.278 Mg m^−^^3^
Monoclinic, *P*2_1_/*n*	Mo *K*α radiation, λ = 0.71073 Å
*a* = 10.790 (2) Å	Cell parameters from 11435 reflections
*b* = 15.600 (3) Å	θ = 2.1–25.5°
*c* = 36.960 (7) Å	µ = 6.75 mm^−^^1^
β = 91.20 (3)°	*T* = 123 K
*V* = 6220 (2) Å^3^	Rod, dark red
*Z* = 4	0.25 × 0.2 × 0.15 mm

### Data collection

**Table:**

Stoe IPDS diffractometer	11435 independent reflections
Radiation source: fine-focus sealed tube	7473 reflections with *I* > 2σ(*I*)
graphite	*R*_int_ = 0.099
rotation scans	θ_max_ = 25.5°, θ_min_ = 2.1°
Absorption correction: analytical (*X-SHAPE*; Stoe & Cie, 2002)	*h* = −13→13
*T*_min_ = 0.057, *T*_max_ = 0.119	*k* = 0→18
36425 measured reflections	*l* = 0→44

### Refinement

**Table:**

Refinement on *F*^2^	Primary atom site location: structure-invariant direct methods
Least-squares matrix: full	Secondary atom site location: difference Fourier map
*R*[*F*^2^ > 2σ(*F*^2^)] = 0.043	Hydrogen site location: inferred from neighbouring sites
*wR*(*F*^2^) = 0.112	H-atom parameters constrained
*S* = 0.84	*w* = 1/[σ^2^(*F*_o_^2^) + (0.0671*P*)^2^] where *P* = (*F*_o_^2^ + 2*F*_c_^2^)/3
11435 reflections	(Δ/σ)_max_ = 0.001
608 parameters	Δρ_max_ = 2.77 e Å^−^^3^
0 restraints	Δρ_min_ = −1.18 e Å^−^^3^

### Special details

**Table:**

Experimental. crystal mounting in perfluorether (T. Kottke, D. Stalke, J. Appl. Crystallogr. 26, 1993, p. 615)
Geometry. All e.s.d.'s (except the e.s.d. in the dihedral angle between two l.s. planes) are estimated using the full covariance matrix. The cell e.s.d.'s are taken into account individually in the estimation of e.s.d.'s in distances, angles and torsion angles; correlations between e.s.d.'s in cell parameters are only used when they are defined by crystal symmetry. An approximate (isotropic) treatment of cell e.s.d.'s is used for estimating e.s.d.'s involving l.s. planes.
Refinement. Refinement of *F*^2^ against ALL reflections. The weighted *R*-factor *wR* and goodness of fit *S* are based on *F*^2^, conventional *R*-factors *R* are based on *F*, with *F* set to zero for negative *F*^2^. The threshold expression of *F*^2^ > σ(*F*^2^) is used only for calculating *R*-factors(gt) *etc*. and is not relevant to the choice of reflections for refinement. *R*-factors based on *F*^2^ are statistically about twice as large as those based on *F*, and *R*- factors based on ALL data will be even larger.

### Fractional atomic coordinates and isotropic or equivalent isotropic displacement parameters (Å^2^)

**Table:**

	*x*	*y*	*z*	*U*_iso_*/*U*_eq_	Occ. (<1)
Sn1	0.86100 (7)	0.23615 (4)	0.907589 (19)	0.04003 (16)	
Sn2	0.98667 (7)	0.39369 (5)	0.931424 (17)	0.03899 (16)	
Sn3	1.02254 (6)	0.31609 (4)	0.854533 (17)	0.03508 (15)	
Sn4	0.71369 (7)	0.30366 (4)	0.846330 (18)	0.03923 (16)	
Sn5	0.69254 (6)	0.37991 (5)	0.923721 (18)	0.04106 (17)	
Sn6	1.03304 (7)	0.50482 (4)	0.86702 (2)	0.04262 (17)	
Sn7	0.87119 (7)	0.43216 (5)	0.809921 (18)	0.04261 (17)	
Sn8	0.66698 (7)	0.48901 (5)	0.856306 (19)	0.04374 (18)	
Sn9	0.82933 (7)	0.54589 (5)	0.91672 (2)	0.04456 (18)	
Rb10	0.57160 (8)	0.40171 (5)	0.75840 (2)	0.03225 (19)	
Rb11	0.66434 (8)	0.09245 (5)	0.09496 (2)	0.03070 (18)	
Rb12	0.81415 (13)	0.45056 (9)	0.01874 (3)	0.0625 (3)	
O1	0.5633 (6)	0.2576 (4)	0.11449 (18)	0.0402 (15)	
O2	0.4199 (6)	0.2343 (4)	0.74399 (16)	0.0358 (14)	
O3	0.6819 (6)	−0.0751 (4)	0.12707 (17)	0.0351 (14)	
O4	0.3954 (7)	0.5552 (4)	0.74943 (18)	0.0398 (15)	
O5	0.6400 (7)	0.5615 (4)	0.72603 (18)	0.0428 (16)	
O6	0.7426 (6)	0.4148 (4)	0.69534 (18)	0.0380 (15)	
O7	0.3089 (6)	0.3882 (4)	0.7689 (2)	0.0423 (16)	
O8	0.8881 (6)	0.0870 (4)	0.05216 (17)	0.0380 (15)	
O9	0.7691 (6)	0.1897 (4)	0.15412 (19)	0.0422 (16)	
O10	0.4441 (6)	−0.0106 (4)	0.10348 (18)	0.0350 (14)	
O11	0.6481 (7)	0.1129 (4)	0.01641 (17)	0.0395 (15)	
O12	0.6651 (7)	0.2526 (4)	0.72188 (18)	0.0429 (16)	
N1	0.4241 (8)	0.1494 (5)	0.0601 (2)	0.0351 (17)	
N2	0.9076 (7)	0.0352 (5)	0.1296 (2)	0.0353 (17)	
N3	0.8485 (13)	0.2982 (8)	0.0671 (3)	0.077 (3)	
H3C	0.9034	0.3114	0.0853	0.115*	
H3D	0.8781	0.2535	0.0541	0.115*	
H3E	0.7744	0.2835	0.0767	0.115*	
N4	0.7481 (11)	0.7826 (7)	0.0683 (3)	0.068 (3)	
H4C	0.7062	0.8276	0.0779	0.102*	
H4D	0.7343	0.7810	0.0439	0.102*	
H4E	0.8307	0.7889	0.0731	0.102*	
N5	0.8929 (11)	0.7596 (9)	−0.0120 (3)	0.076 (3)	
N6	0.5861 (13)	0.4400 (7)	0.0693 (4)	0.085 (4)	
H6C	0.6234	0.3966	0.0818	0.127*	
H6D	0.5582	0.4200	0.0474	0.127*	
H6E	0.5211	0.4604	0.0820	0.127*	
N7	0.6387 (13)	0.6110 (8)	0.0138 (3)	0.080 (4)	
H7C	0.5573	0.5956	0.0138	0.121*	
H7D	0.6738	0.5923	−0.0070	0.121*	
H7E	0.6449	0.6691	0.0151	0.121*	
C1	0.8604 (10)	0.0730 (7)	0.0140 (3)	0.043 (2)	
H1A	0.8298	0.0138	0.0103	0.052*	
H1B	0.9366	0.0804	−0.0001	0.052*	
C2	0.9754 (10)	0.0273 (7)	0.0661 (3)	0.044 (2)	
H2A	1.0512	0.0286	0.0514	0.053*	
H2B	0.9403	−0.0313	0.0648	0.053*	
C3	0.4776 (11)	0.6220 (6)	0.7593 (3)	0.045 (3)	
H3A	0.5229	0.6066	0.7820	0.055*	
H3B	0.4301	0.6752	0.7638	0.055*	
C4	0.7647 (9)	0.1352 (7)	0.0014 (2)	0.037 (2)	
H4A	0.7886	0.1938	0.0091	0.045*	
H4B	0.7582	0.1343	−0.0253	0.045*	
C5	0.7776 (9)	−0.0840 (7)	0.1535 (3)	0.042 (2)	
H5A	0.7834	−0.1446	0.1612	0.050*	
H5B	0.7581	−0.0489	0.1749	0.050*	
C6	0.5676 (11)	0.6370 (7)	0.7299 (3)	0.048 (3)	
H6A	0.5227	0.6500	0.7070	0.058*	
H6B	0.6218	0.6862	0.7362	0.058*	
C7	0.2302 (9)	0.4614 (7)	0.7664 (3)	0.042 (2)	
H7A	0.1600	0.4551	0.7830	0.050*	
H7B	0.1965	0.4673	0.7414	0.050*	
C8	0.3563 (9)	0.0031 (7)	0.0747 (3)	0.043 (2)	
H8A	0.2810	−0.0315	0.0788	0.051*	
H8B	0.3921	−0.0150	0.0514	0.051*	
C9	0.3235 (9)	0.0957 (7)	0.0731 (3)	0.042 (2)	
H9A	0.2500	0.1032	0.0569	0.050*	
H9B	0.3007	0.1152	0.0976	0.050*	
C10	0.4330 (10)	0.2605 (7)	0.1088 (2)	0.039 (2)	
H10A	0.4016	0.3183	0.1148	0.047*	
H10B	0.3926	0.2185	0.1248	0.047*	
C11	0.3289 (10)	0.2381 (7)	0.7713 (3)	0.042 (2)	
H11A	0.3695	0.2472	0.7953	0.051*	
H11B	0.2823	0.1835	0.7720	0.051*	
C12	0.5558 (10)	0.1729 (7)	0.0061 (3)	0.043 (2)	
H12A	0.5516	0.1780	−0.0206	0.052*	
H12B	0.5764	0.2299	0.0163	0.052*	
C13	0.5999 (10)	0.2839 (7)	0.1511 (3)	0.044 (2)	
H13A	0.5563	0.2479	0.1688	0.053*	
H13B	0.5752	0.3443	0.1550	0.053*	
C14	0.4731 (10)	−0.0986 (6)	0.1086 (3)	0.046 (3)	
H14A	0.5045	−0.1228	0.0858	0.055*	
H14B	0.3971	−0.1303	0.1149	0.055*	
C15	0.2431 (9)	0.3100 (7)	0.7630 (3)	0.040 (2)	
H15A	0.2135	0.3065	0.7375	0.048*	
H15B	0.1703	0.3074	0.7788	0.048*	
C16	0.7234 (10)	0.5677 (7)	0.6960 (3)	0.041 (2)	
H16A	0.7707	0.6220	0.6973	0.050*	
H16B	0.6759	0.5665	0.6728	0.050*	
C17	0.7518 (10)	0.2623 (7)	0.6930 (3)	0.046 (3)	
H17A	0.7067	0.2642	0.6694	0.055*	
H17B	0.8093	0.2129	0.6928	0.055*	
C18	0.7343 (10)	0.2755 (7)	0.1571 (3)	0.042 (2)	
H18A	0.7784	0.3102	0.1390	0.050*	
H18B	0.7575	0.2973	0.1814	0.050*	
C19	0.5680 (10)	−0.1097 (6)	0.1379 (3)	0.044 (2)	
H19A	0.5406	−0.0804	0.1601	0.053*	
H19B	0.5783	−0.1714	0.1434	0.053*	
C20	0.4024 (12)	0.2398 (7)	0.0699 (3)	0.052 (3)	
H20A	0.3141	0.2538	0.0649	0.062*	
H20B	0.4529	0.2768	0.0542	0.062*	
C21	1.0067 (9)	0.0497 (7)	0.1044 (3)	0.042 (2)	
H21A	1.0798	0.0157	0.1123	0.050*	
H21B	1.0305	0.1110	0.1054	0.050*	
C22	0.5137 (10)	0.1715 (6)	0.7512 (3)	0.043 (2)	
H22A	0.4756	0.1140	0.7531	0.052*	
H22B	0.5572	0.1846	0.7744	0.052*	
C23	0.8992 (9)	0.1786 (7)	0.1595 (3)	0.045 (3)	
H23A	0.9272	0.2097	0.1816	0.054*	
H23B	0.9438	0.2024	0.1387	0.054*	
C24	0.8224 (10)	0.3427 (7)	0.6986 (3)	0.044 (2)	
H24A	0.8624	0.3423	0.7229	0.052*	
H24B	0.8883	0.3467	0.6804	0.052*	
C25	0.9278 (9)	0.0843 (7)	0.1635 (3)	0.044 (2)	
H25A	1.0153	0.0775	0.1716	0.053*	
H25B	0.8749	0.0598	0.1825	0.053*	
C26	0.3043 (10)	0.5380 (7)	0.7763 (3)	0.044 (2)	
H26A	0.2488	0.5881	0.7786	0.053*	
H26B	0.3462	0.5285	0.8001	0.053*	
C27	0.4310 (10)	0.1429 (7)	0.0200 (3)	0.045 (2)	
H27A	0.3642	0.1780	0.0088	0.055*	
H27B	0.4172	0.0825	0.0127	0.055*	
C28	0.8094 (9)	0.4932 (6)	0.6982 (3)	0.040 (2)	
H28A	0.8698	0.4968	0.6785	0.048*	
H28B	0.8560	0.4948	0.7216	0.048*	
C29	0.8976 (9)	−0.0564 (7)	0.1388 (3)	0.042 (2)	
H29A	0.9637	−0.0705	0.1568	0.050*	
H29B	0.9135	−0.0903	0.1168	0.050*	
C30	0.6027 (10)	0.1724 (6)	0.7214 (3)	0.044 (2)	
H30A	0.6632	0.1252	0.7245	0.053*	
H30B	0.5583	0.1645	0.6979	0.053*	
Rb13	0.6569 (4)	0.6145 (3)	0.10223 (8)	0.0558 (12)	0.74 (2)
Rb14	0.6416 (11)	0.6289 (8)	0.1185 (15)	0.130 (9)	0.26 (2)

### Atomic displacement parameters (Å^2^)

**Table:**

	*U*^11^	*U*^22^	*U*^33^	*U*^12^	*U*^13^	*U*^23^
Sn1	0.0417 (4)	0.0373 (4)	0.0413 (4)	0.0003 (3)	0.0037 (3)	0.0097 (3)
Sn2	0.0417 (4)	0.0457 (4)	0.0294 (3)	0.0058 (3)	−0.0039 (3)	−0.0026 (3)
Sn3	0.0406 (4)	0.0334 (3)	0.0314 (3)	0.0037 (3)	0.0060 (3)	0.0004 (3)
Sn4	0.0514 (4)	0.0332 (3)	0.0328 (3)	−0.0058 (3)	−0.0047 (3)	0.0010 (3)
Sn5	0.0360 (4)	0.0540 (4)	0.0335 (3)	0.0033 (3)	0.0056 (3)	−0.0003 (3)
Sn6	0.0515 (4)	0.0342 (3)	0.0423 (4)	−0.0078 (3)	0.0037 (3)	−0.0009 (3)
Sn7	0.0376 (4)	0.0585 (4)	0.0316 (3)	−0.0021 (3)	−0.0013 (3)	0.0103 (3)
Sn8	0.0518 (4)	0.0409 (4)	0.0381 (4)	0.0132 (3)	−0.0079 (3)	−0.0045 (3)
Sn9	0.0474 (4)	0.0405 (4)	0.0456 (4)	0.0082 (3)	−0.0046 (3)	−0.0126 (3)
Rb10	0.0341 (5)	0.0319 (4)	0.0307 (4)	0.0001 (3)	0.0001 (3)	0.0001 (3)
Rb11	0.0337 (5)	0.0319 (4)	0.0266 (4)	0.0006 (3)	0.0018 (3)	−0.0005 (3)
Rb12	0.0672 (8)	0.0778 (8)	0.0427 (6)	−0.0158 (6)	0.0066 (5)	0.0015 (6)
O1	0.039 (4)	0.044 (4)	0.038 (4)	0.001 (3)	0.007 (3)	−0.002 (3)
O2	0.040 (4)	0.038 (3)	0.029 (3)	0.007 (3)	0.000 (3)	0.008 (3)
O3	0.027 (3)	0.045 (4)	0.033 (3)	0.007 (3)	0.000 (3)	0.006 (3)
O4	0.050 (4)	0.033 (3)	0.036 (4)	−0.003 (3)	0.000 (3)	−0.003 (3)
O5	0.052 (4)	0.038 (4)	0.038 (4)	0.002 (3)	0.008 (3)	0.001 (3)
O6	0.033 (3)	0.044 (4)	0.037 (4)	−0.004 (3)	0.005 (3)	−0.001 (3)
O7	0.037 (4)	0.035 (4)	0.055 (4)	−0.002 (3)	0.008 (3)	−0.008 (3)
O8	0.039 (4)	0.049 (4)	0.026 (3)	0.004 (3)	0.001 (3)	−0.006 (3)
O9	0.041 (4)	0.045 (4)	0.042 (4)	−0.005 (3)	0.003 (3)	−0.015 (3)
O10	0.028 (3)	0.036 (3)	0.041 (4)	0.001 (3)	0.000 (3)	0.003 (3)
O11	0.049 (4)	0.042 (4)	0.027 (3)	−0.002 (3)	0.002 (3)	0.004 (3)
O12	0.055 (4)	0.032 (4)	0.042 (4)	−0.002 (3)	0.010 (3)	−0.004 (3)
N1	0.042 (5)	0.037 (4)	0.027 (4)	0.005 (3)	−0.001 (3)	0.001 (3)
N2	0.030 (4)	0.047 (5)	0.028 (4)	0.003 (3)	−0.005 (3)	−0.001 (3)
N3	0.101 (9)	0.082 (8)	0.047 (6)	−0.004 (7)	−0.002 (6)	−0.017 (6)
N4	0.063 (7)	0.072 (7)	0.068 (7)	0.018 (6)	−0.014 (5)	−0.019 (6)
N5	0.058 (7)	0.110 (10)	0.061 (7)	0.032 (7)	0.007 (5)	0.014 (7)
N6	0.095 (10)	0.055 (7)	0.107 (10)	−0.005 (6)	0.041 (8)	−0.005 (7)
N7	0.085 (9)	0.081 (8)	0.076 (8)	0.012 (7)	0.019 (7)	0.011 (7)
C1	0.043 (6)	0.055 (6)	0.031 (5)	−0.008 (5)	0.010 (4)	−0.014 (4)
C2	0.044 (6)	0.052 (6)	0.037 (5)	0.004 (5)	0.006 (4)	−0.004 (5)
C3	0.065 (7)	0.031 (5)	0.040 (5)	0.000 (5)	−0.014 (5)	−0.007 (4)
C4	0.030 (5)	0.060 (6)	0.022 (4)	−0.004 (4)	−0.002 (4)	−0.005 (4)
C5	0.032 (5)	0.048 (6)	0.045 (6)	0.008 (4)	−0.002 (4)	0.015 (5)
C6	0.070 (8)	0.033 (5)	0.041 (6)	−0.002 (5)	−0.013 (5)	−0.003 (4)
C7	0.035 (5)	0.050 (6)	0.041 (5)	0.007 (4)	0.000 (4)	−0.011 (5)
C8	0.032 (5)	0.054 (6)	0.041 (5)	−0.006 (4)	−0.012 (4)	0.005 (5)
C9	0.031 (5)	0.051 (6)	0.041 (5)	−0.001 (4)	−0.006 (4)	0.003 (5)
C10	0.044 (6)	0.044 (5)	0.029 (5)	0.011 (4)	0.003 (4)	0.000 (4)
C11	0.041 (6)	0.053 (6)	0.033 (5)	−0.007 (5)	0.002 (4)	0.003 (4)
C12	0.056 (7)	0.046 (6)	0.026 (5)	0.006 (5)	0.000 (4)	0.003 (4)
C13	0.052 (6)	0.046 (6)	0.035 (5)	−0.001 (5)	0.008 (5)	−0.014 (4)
C14	0.037 (6)	0.031 (5)	0.070 (7)	−0.002 (4)	−0.010 (5)	0.006 (5)
C15	0.035 (5)	0.049 (6)	0.037 (5)	−0.010 (4)	0.011 (4)	−0.007 (4)
C16	0.051 (6)	0.041 (5)	0.032 (5)	−0.011 (5)	0.003 (4)	0.004 (4)
C17	0.049 (6)	0.047 (6)	0.043 (6)	0.015 (5)	0.016 (5)	−0.012 (5)
C18	0.049 (6)	0.042 (6)	0.034 (5)	−0.008 (5)	0.002 (4)	−0.010 (4)
C19	0.045 (6)	0.035 (5)	0.052 (6)	−0.001 (4)	0.016 (5)	0.012 (5)
C20	0.069 (8)	0.042 (6)	0.045 (6)	0.016 (5)	0.014 (5)	0.003 (5)
C21	0.031 (5)	0.063 (7)	0.030 (5)	0.005 (5)	−0.001 (4)	−0.008 (4)
C22	0.050 (6)	0.039 (5)	0.040 (5)	−0.005 (4)	−0.008 (5)	0.003 (4)
C23	0.033 (5)	0.063 (7)	0.039 (5)	−0.015 (5)	0.003 (4)	−0.012 (5)
C24	0.041 (6)	0.053 (6)	0.036 (5)	0.004 (5)	0.002 (4)	−0.008 (4)
C25	0.026 (5)	0.072 (7)	0.034 (5)	−0.007 (5)	−0.002 (4)	−0.007 (5)
C26	0.054 (7)	0.047 (6)	0.031 (5)	0.017 (5)	0.003 (4)	−0.005 (4)
C27	0.051 (6)	0.050 (6)	0.035 (5)	0.014 (5)	−0.003 (5)	0.000 (5)
C28	0.038 (5)	0.044 (6)	0.038 (5)	−0.012 (4)	0.002 (4)	0.004 (4)
C29	0.031 (5)	0.057 (6)	0.037 (5)	0.015 (4)	−0.006 (4)	0.009 (5)
C30	0.049 (6)	0.034 (5)	0.049 (6)	0.002 (4)	−0.015 (5)	−0.004 (4)
Rb13	0.0371 (13)	0.0645 (18)	0.066 (2)	−0.0003 (10)	−0.0051 (8)	−0.0027 (10)
Rb14	0.035 (4)	0.075 (5)	0.28 (3)	0.011 (3)	−0.002 (9)	−0.026 (10)

### Geometric parameters (Å, °)

**Table:**

Sn1—Sn3	2.9295 (12)	N6—H6C	0.9100
Sn1—Sn2	2.9334 (11)	N6—H6D	0.9100
Sn1—Sn4	2.9344 (12)	N6—H6E	0.9100
Sn1—Sn5	2.9553 (11)	N7—H7C	0.9100
Sn2—Sn9	2.9623 (11)	N7—H7D	0.9100
Sn2—Sn6	2.9954 (11)	N7—H7E	0.9100
Sn2—Sn3	3.1206 (11)	C1—C4	1.484 (15)
Sn2—Sn5	3.1877 (12)	C1—H1A	0.9900
Sn2—Rb12^i^	3.7080 (16)	C1—H1B	0.9900
Sn2—Rb12^ii^	3.8625 (17)	C2—C21	1.490 (13)
Sn2—Rb13^i^	4.069 (5)	C2—H2A	0.9900
Sn3—Sn7	2.9243 (11)	C2—H2B	0.9900
Sn3—Sn6	2.9819 (11)	C3—C6	1.491 (17)
Sn3—Rb14^i^	3.835 (16)	C3—H3A	0.9900
Sn3—Rb13^i^	3.932 (3)	C3—H3B	0.9900
Sn4—Sn8	2.9594 (11)	C4—H4A	0.9900
Sn4—Sn7	2.9687 (11)	C4—H4B	0.9900
Sn4—Sn5	3.1106 (11)	C5—C29	1.478 (14)
Sn4—Rb10	3.8784 (14)	C5—H5A	0.9900
Sn4—Rb14^iii^	4.21 (2)	C5—H5B	0.9900
Sn5—Sn9	2.9942 (12)	C6—H6A	0.9900
Sn5—Sn8	3.0252 (11)	C6—H6B	0.9900
Sn5—Rb13^iii^	3.872 (4)	C7—C26	1.479 (15)
Sn5—Rb12^ii^	3.8829 (17)	C7—H7A	0.9900
Sn5—Rb14^iii^	3.90 (2)	C7—H7B	0.9900
Sn6—Sn7	2.9382 (13)	C8—C9	1.489 (15)
Sn6—Sn9	2.9642 (14)	C8—H8A	0.9900
Sn6—Sn8	3.9689 (14)	C8—H8B	0.9900
Sn6—Rb13^i^	3.974 (5)	C9—H9A	0.9900
Sn6—Rb14^i^	4.109 (11)	C9—H9B	0.9900
Sn7—Sn8	2.9561 (13)	C10—C20	1.503 (15)
Sn7—Rb10	3.7474 (15)	C10—H10A	0.9900
Sn8—Rb14^iii^	3.93 (2)	C10—H10B	0.9900
Sn8—Rb10	3.9814 (14)	C11—C15	1.483 (15)
Sn8—Rb13^iii^	4.171 (4)	C11—H11A	0.9900
Sn9—Rb12^ii^	4.0597 (16)	C11—H11B	0.9900
Rb10—O5	2.869 (7)	C12—C27	1.525 (16)
Rb10—O7	2.876 (7)	C12—H12A	0.9900
Rb10—O12	2.882 (7)	C12—H12B	0.9900
Rb10—O6	3.010 (6)	C13—C18	1.467 (15)
Rb10—O4	3.071 (7)	C13—H13A	0.9900
Rb10—O2	3.122 (7)	C13—H13B	0.9900
Rb11—O9	2.873 (7)	C14—C19	1.483 (15)
Rb11—O3	2.875 (6)	C14—H14A	0.9900
Rb11—O10	2.891 (6)	C14—H14B	0.9900
Rb11—O1	2.896 (7)	C15—H15A	0.9900
Rb11—O8	2.915 (7)	C15—H15B	0.9900
Rb11—O11	2.922 (6)	C16—C28	1.488 (15)
Rb11—N1	3.004 (8)	C16—H16A	0.9900
Rb11—N2	3.032 (8)	C16—H16B	0.9900
Rb12—N3	2.992 (12)	C17—C24	1.479 (15)
Rb12—Sn2^i^	3.7080 (16)	C17—H17A	0.9900
Rb12—Sn2^iv^	3.8625 (17)	C17—H17B	0.9900
Rb12—Sn5^iv^	3.8829 (17)	C18—H18A	0.9900
Rb12—Sn9^iv^	4.0597 (16)	C18—H18B	0.9900
O1—C10	1.418 (12)	C19—H19A	0.9900
O1—C13	1.460 (12)	C19—H19B	0.9900
O2—C11	1.425 (12)	C20—H20A	0.9900
O2—C22	1.430 (12)	C20—H20B	0.9900
O3—C19	1.407 (12)	C21—H21A	0.9900
O3—C5	1.412 (11)	C21—H21B	0.9900
O4—C3	1.413 (12)	C22—C30	1.477 (16)
O4—C26	1.437 (12)	C22—H22A	0.9900
O5—C6	1.421 (13)	C22—H22B	0.9900
O5—C16	1.448 (12)	C23—C25	1.511 (16)
O6—C24	1.420 (12)	C23—H23A	0.9900
O6—C28	1.423 (12)	C23—H23B	0.9900
O7—C7	1.426 (12)	C24—H24A	0.9900
O7—C15	1.426 (12)	C24—H24B	0.9900
O8—C2	1.414 (12)	C25—H25A	0.9900
O8—C1	1.450 (11)	C25—H25B	0.9900
O9—C18	1.396 (12)	C26—H26A	0.9900
O9—C23	1.425 (13)	C26—H26B	0.9900
O10—C14	1.421 (11)	C27—H27A	0.9900
O10—C8	1.426 (11)	C27—H27B	0.9900
O11—C12	1.413 (12)	C28—H28A	0.9900
O11—C4	1.428 (12)	C28—H28B	0.9900
O12—C30	1.421 (12)	C29—H29A	0.9900
O12—C17	1.442 (12)	C29—H29B	0.9900
N1—C9	1.460 (13)	C30—H30A	0.9900
N1—C20	1.476 (13)	C30—H30B	0.9900
N1—C27	1.489 (12)	Rb13—Sn5^iii^	3.872 (4)
N2—C21	1.453 (13)	Rb13—Sn3^i^	3.932 (3)
N2—C29	1.473 (13)	Rb13—Sn6^i^	3.974 (5)
N2—C25	1.480 (12)	Rb13—Sn2^i^	4.069 (5)
N3—H3C	0.9100	Rb13—Sn8^iii^	4.171 (4)
N3—H3D	0.9100	Rb14—Sn3^i^	3.835 (15)
N3—H3E	0.9100	Rb14—Sn5^iii^	3.90 (2)
N4—Rb13	3.077 (13)	Rb14—Sn8^iii^	3.933 (19)
N4—H4C	0.9100	Rb14—Sn6^i^	4.109 (11)
N4—H4D	0.9100	Rb14—Sn4^iii^	4.21 (2)
N4—H4E	0.9100		
			
Sn3—Sn1—Sn2	64.32 (3)	C18—O9—C23	111.8 (8)
Sn3—Sn1—Sn4	69.57 (3)	C18—O9—Rb11	117.6 (6)
Sn2—Sn1—Sn4	99.82 (3)	C23—O9—Rb11	114.4 (5)
Sn3—Sn1—Sn5	100.90 (3)	C14—O10—C8	112.7 (8)
Sn2—Sn1—Sn5	65.55 (3)	C14—O10—Rb11	111.9 (5)
Sn4—Sn1—Sn5	63.76 (3)	C8—O10—Rb11	111.7 (5)
Sn1—Sn2—Sn9	110.91 (3)	C12—O11—C4	110.9 (7)
Sn1—Sn2—Sn6	109.27 (3)	C12—O11—Rb11	111.6 (5)
Sn9—Sn2—Sn6	59.67 (3)	C4—O11—Rb11	112.1 (5)
Sn1—Sn2—Sn3	57.78 (3)	C30—O12—C17	113.4 (8)
Sn9—Sn2—Sn3	103.03 (3)	C30—O12—Rb10	123.1 (6)
Sn6—Sn2—Sn3	58.32 (3)	C17—O12—Rb10	120.1 (5)
Sn1—Sn2—Sn5	57.56 (3)	C9—N1—C20	110.1 (8)
Sn9—Sn2—Sn5	58.13 (3)	C9—N1—C27	109.9 (8)
Sn6—Sn2—Sn5	98.66 (4)	C20—N1—C27	108.7 (8)
Sn3—Sn2—Sn5	91.99 (4)	C9—N1—Rb11	109.3 (5)
Sn1—Sn2—Rb12^i^	163.26 (3)	C20—N1—Rb11	108.5 (7)
Sn9—Sn2—Rb12^i^	83.68 (4)	C27—N1—Rb11	110.2 (6)
Sn6—Sn2—Rb12^i^	84.88 (3)	C21—N2—C29	111.0 (8)
Sn3—Sn2—Rb12^i^	128.68 (4)	C21—N2—C25	111.5 (8)
Sn5—Sn2—Rb12^i^	130.86 (4)	C29—N2—C25	108.5 (8)
Sn1—Sn2—Rb12^ii^	102.44 (3)	C21—N2—Rb11	108.9 (5)
Sn9—Sn2—Rb12^ii^	71.63 (3)	C29—N2—Rb11	108.4 (5)
Sn6—Sn2—Rb12^ii^	128.50 (4)	C25—N2—Rb11	108.4 (6)
Sn3—Sn2—Rb12^ii^	157.10 (3)	Rb12—N3—H3C	109.5
Sn5—Sn2—Rb12^ii^	66.03 (4)	Rb12—N3—H3D	109.5
Rb12^i^—Sn2—Rb12^ii^	73.68 (4)	H3C—N3—H3D	109.5
Sn1—Sn2—Rb13^i^	108.41 (7)	Rb12—N3—H3E	109.5
Sn9—Sn2—Rb13^i^	120.72 (7)	H3C—N3—H3E	109.5
Sn6—Sn2—Rb13^i^	66.46 (6)	H3D—N3—H3E	109.5
Sn3—Sn2—Rb13^i^	64.74 (5)	Rb13—N4—H4C	109.5
Sn5—Sn2—Rb13^i^	156.38 (5)	Rb13—N4—H4D	109.5
Rb12^i^—Sn2—Rb13^i^	68.33 (6)	H4C—N4—H4D	109.5
Rb12^ii^—Sn2—Rb13^i^	137.55 (5)	Rb13—N4—H4E	109.5
Sn7—Sn3—Sn1	107.93 (3)	H4C—N4—H4E	109.5
Sn7—Sn3—Sn6	59.66 (3)	H4D—N4—H4E	109.5
Sn1—Sn3—Sn6	109.75 (3)	H6C—N6—H6D	109.5
Sn7—Sn3—Sn2	101.23 (3)	H6C—N6—H6E	109.5
Sn1—Sn3—Sn2	57.90 (3)	H6D—N6—H6E	109.5
Sn6—Sn3—Sn2	58.74 (2)	H7C—N7—H7D	109.5
Sn7—Sn3—Rb14^i^	121.4 (4)	H7C—N7—H7E	109.5
Sn1—Sn3—Rb14^i^	119.6 (8)	H7D—N7—H7E	109.5
Sn6—Sn3—Rb14^i^	72.9 (2)	O8—C1—C4	109.5 (8)
Sn2—Sn3—Rb14^i^	79.1 (8)	O8—C1—Rb11	46.7 (4)
Sn7—Sn3—Rb13^i^	122.33 (7)	C4—C1—Rb11	78.2 (5)
Sn1—Sn3—Rb13^i^	112.08 (5)	O8—C1—H1A	109.8
Sn6—Sn3—Rb13^i^	68.59 (8)	C4—C1—H1A	109.8
Sn2—Sn3—Rb13^i^	69.38 (7)	Rb11—C1—H1A	89.7
Rb14^i^—Sn3—Rb13^i^	9.7 (7)	O8—C1—H1B	109.8
Sn1—Sn4—Sn8	110.21 (3)	C4—C1—H1B	109.8
Sn1—Sn4—Sn7	106.63 (3)	Rb11—C1—H1B	155.4
Sn8—Sn4—Sn7	59.82 (3)	H1A—C1—H1B	108.2
Sn1—Sn4—Sn5	58.45 (3)	O8—C2—C21	109.1 (8)
Sn8—Sn4—Sn5	59.73 (2)	O8—C2—Rb11	47.1 (4)
Sn7—Sn4—Sn5	102.26 (3)	C21—C2—Rb11	81.4 (6)
Sn1—Sn4—Rb10	170.49 (3)	O8—C2—H2A	109.9
Sn8—Sn4—Rb10	69.74 (2)	C21—C2—H2A	109.9
Sn7—Sn4—Rb10	64.78 (3)	Rb11—C2—H2A	156.9
Sn5—Sn4—Rb10	125.76 (3)	O8—C2—H2B	109.9
Sn1—Sn4—Rb14^iii^	109.5 (7)	C21—C2—H2B	109.9
Sn8—Sn4—Rb14^iii^	63.80 (18)	Rb11—C2—H2B	85.3
Sn7—Sn4—Rb14^iii^	120.3 (3)	H2A—C2—H2B	108.3
Sn5—Sn4—Rb14^iii^	62.3 (7)	O4—C3—C6	110.0 (8)
Rb10—Sn4—Rb14^iii^	79.2 (7)	O4—C3—Rb10	57.7 (5)
Sn1—Sn5—Sn9	109.42 (3)	C6—C3—Rb10	87.4 (6)
Sn1—Sn5—Sn8	107.85 (3)	O4—C3—H3A	109.7
Sn9—Sn5—Sn8	58.60 (3)	C6—C3—H3A	109.7
Sn1—Sn5—Sn4	57.79 (3)	Rb10—C3—H3A	68.9
Sn9—Sn5—Sn4	101.86 (3)	O4—C3—H3B	109.7
Sn8—Sn5—Sn4	57.65 (3)	C6—C3—H3B	109.7
Sn1—Sn5—Sn2	56.90 (2)	Rb10—C3—H3B	162.2
Sn9—Sn5—Sn2	57.16 (2)	H3A—C3—H3B	108.2
Sn8—Sn5—Sn2	96.26 (4)	O11—C4—C1	109.4 (8)
Sn4—Sn5—Sn2	90.91 (4)	O11—C4—Rb11	47.0 (4)
Sn1—Sn5—Rb13^iii^	124.54 (8)	C1—C4—Rb11	78.7 (5)
Sn9—Sn5—Rb13^iii^	116.00 (7)	O11—C4—H4A	109.8
Sn8—Sn5—Rb13^iii^	73.25 (5)	C1—C4—H4A	109.8
Sn4—Sn5—Rb13^iii^	82.57 (7)	Rb11—C4—H4A	88.7
Sn2—Sn5—Rb13^iii^	169.45 (5)	O11—C4—H4B	109.8
Sn1—Sn5—Rb12^ii^	101.55 (4)	C1—C4—H4B	109.8
Sn9—Sn5—Rb12^ii^	71.02 (3)	Rb11—C4—H4B	156.0
Sn8—Sn5—Rb12^ii^	127.48 (4)	H4A—C4—H4B	108.2
Sn4—Sn5—Rb12^ii^	155.55 (3)	O3—C5—C29	110.6 (8)
Sn2—Sn5—Rb12^ii^	65.36 (4)	O3—C5—Rb11	45.1 (4)
Rb13^iii^—Sn5—Rb12^ii^	121.77 (7)	C29—C5—Rb11	81.4 (5)
Sn1—Sn5—Rb14^iii^	117.4 (5)	O3—C5—H5A	109.5
Sn9—Sn5—Rb14^iii^	116.7 (3)	C29—C5—H5A	109.5
Sn8—Sn5—Rb14^iii^	67.8 (6)	Rb11—C5—H5A	154.1
Sn4—Sn5—Rb14^iii^	72.7 (7)	O3—C5—H5B	109.5
Sn2—Sn5—Rb14^iii^	161.5 (8)	C29—C5—H5B	109.5
Rb13^iii^—Sn5—Rb14^iii^	9.8 (7)	Rb11—C5—H5B	89.0
Rb12^ii^—Sn5—Rb14^iii^	131.6 (7)	H5A—C5—H5B	108.1
Sn7—Sn6—Sn9	95.23 (3)	O5—C6—C3	108.2 (8)
Sn7—Sn6—Sn3	59.20 (2)	O5—C6—H6A	110.1
Sn9—Sn6—Sn3	106.44 (3)	C3—C6—H6A	110.1
Sn7—Sn6—Sn2	103.94 (3)	O5—C6—H6B	110.1
Sn9—Sn6—Sn2	59.61 (3)	C3—C6—H6B	110.1
Sn3—Sn6—Sn2	62.94 (2)	H6A—C6—H6B	108.4
Sn7—Sn6—Sn8	47.87 (3)	O7—C7—C26	108.2 (8)
Sn9—Sn6—Sn8	47.62 (3)	O7—C7—H7A	110.1
Sn3—Sn6—Sn8	83.61 (2)	C26—C7—H7A	110.1
Sn2—Sn6—Sn8	82.00 (3)	O7—C7—H7B	110.1
Sn7—Sn6—Rb13^i^	120.59 (7)	C26—C7—H7B	110.1
Sn9—Sn6—Rb13^i^	123.69 (6)	H7A—C7—H7B	108.4
Sn3—Sn6—Rb13^i^	67.09 (6)	O10—C8—C9	109.1 (8)
Sn2—Sn6—Rb13^i^	69.83 (5)	O10—C8—Rb11	47.1 (4)
Sn8—Sn6—Rb13^i^	146.05 (6)	C9—C8—Rb11	81.5 (5)
Sn7—Sn6—Rb14^i^	113.0 (6)	O10—C8—H8A	109.9
Sn9—Sn6—Rb14^i^	132.2 (7)	C9—C8—H8A	109.9
Sn3—Sn6—Rb14^i^	63.2 (3)	Rb11—C8—H8A	156.9
Sn2—Sn6—Rb14^i^	76.1 (6)	O10—C8—H8B	109.9
Sn8—Sn6—Rb14^i^	145.81 (19)	C9—C8—H8B	109.9
Rb13^i^—Sn6—Rb14^i^	9.3 (7)	Rb11—C8—H8B	85.3
Sn3—Sn7—Sn6	61.15 (3)	H8A—C8—H8B	108.3
Sn3—Sn7—Sn8	105.84 (3)	N1—C9—C8	113.1 (8)
Sn6—Sn7—Sn8	84.65 (3)	N1—C9—Rb11	49.1 (4)
Sn3—Sn7—Sn4	69.18 (3)	C8—C9—Rb11	75.3 (5)
Sn6—Sn7—Sn4	105.80 (3)	N1—C9—H9A	109.0
Sn8—Sn7—Sn4	59.93 (3)	C8—C9—H9A	109.0
Sn3—Sn7—Rb10	132.34 (3)	Rb11—C9—H9A	154.3
Sn6—Sn7—Rb10	155.13 (3)	N1—C9—H9B	109.0
Sn8—Sn7—Rb10	71.77 (3)	C8—C9—H9B	109.0
Sn4—Sn7—Rb10	69.44 (3)	Rb11—C9—H9B	93.9
Sn3—Sn7—Sn9	78.97 (3)	H9A—C9—H9B	107.8
Sn6—Sn7—Sn9	42.61 (2)	O1—C10—C20	109.4 (8)
Sn8—Sn7—Sn9	42.29 (3)	O1—C10—Rb11	47.3 (4)
Sn4—Sn7—Sn9	78.07 (3)	C20—C10—Rb11	81.4 (6)
Rb10—Sn7—Sn9	114.02 (3)	O1—C10—H10A	109.8
Sn9—Sn8—Sn7	95.24 (3)	C20—C10—H10A	109.8
Sn9—Sn8—Sn4	106.78 (3)	Rb11—C10—H10A	157.0
Sn7—Sn8—Sn4	60.25 (2)	O1—C10—H10B	109.8
Sn9—Sn8—Sn5	60.17 (3)	C20—C10—H10B	109.8
Sn7—Sn8—Sn5	104.64 (3)	Rb11—C10—H10B	85.4
Sn4—Sn8—Sn5	62.62 (3)	H10A—C10—H10B	108.2
Sn9—Sn8—Rb14^iii^	117.0 (8)	O2—C11—C15	108.7 (8)
Sn7—Sn8—Rb14^iii^	129.9 (5)	O2—C11—Rb10	55.7 (5)
Sn4—Sn8—Rb14^iii^	73.7 (3)	C15—C11—Rb10	83.8 (6)
Sn5—Sn8—Rb14^iii^	66.7 (6)	O2—C11—H11A	110.0
Sn9—Sn8—Sn6	48.01 (3)	C15—C11—H11A	110.0
Sn7—Sn8—Sn6	47.48 (3)	Rb10—C11—H11A	73.4
Sn4—Sn8—Sn6	84.34 (2)	O2—C11—H11B	110.0
Sn5—Sn8—Sn6	83.08 (3)	C15—C11—H11B	110.0
Rb14^iii^—Sn8—Sn6	148.3 (4)	Rb10—C11—H11B	163.7
Sn9—Sn8—Rb10	158.47 (3)	H11A—C11—H11B	108.3
Sn7—Sn8—Rb10	63.38 (3)	O11—C12—C27	109.1 (8)
Sn4—Sn8—Rb10	66.05 (2)	O11—C12—Rb11	47.5 (4)
Sn5—Sn8—Rb10	124.99 (3)	C27—C12—Rb11	82.0 (5)
Rb14^iii^—Sn8—Rb10	81.4 (8)	O11—C12—H12A	109.9
Sn6—Sn8—Rb10	110.47 (4)	C27—C12—H12A	109.9
Sn9—Sn8—Rb13^iii^	109.12 (5)	Rb11—C12—H12A	157.4
Sn7—Sn8—Rb13^iii^	137.51 (8)	O11—C12—H12B	109.9
Sn4—Sn8—Rb13^iii^	79.26 (8)	C27—C12—H12B	109.9
Sn5—Sn8—Rb13^iii^	62.75 (6)	Rb11—C12—H12B	84.3
Rb14^iii^—Sn8—Rb13^iii^	8.8 (7)	H12A—C12—H12B	108.3
Sn6—Sn8—Rb13^iii^	145.82 (5)	O1—C13—C18	111.2 (8)
Rb10—Sn8—Rb13^iii^	89.94 (5)	O1—C13—Rb11	46.1 (4)
Sn8—Sn9—Sn2	103.12 (3)	C18—C13—Rb11	79.4 (5)
Sn8—Sn9—Sn6	84.37 (3)	O1—C13—H13A	109.4
Sn2—Sn9—Sn6	60.72 (3)	C18—C13—H13A	109.4
Sn8—Sn9—Sn5	61.23 (3)	Rb11—C13—H13A	90.7
Sn2—Sn9—Sn5	64.71 (3)	O1—C13—H13B	109.4
Sn6—Sn9—Sn5	103.88 (3)	C18—C13—H13B	109.4
Sn8—Sn9—Rb12^ii^	123.97 (4)	Rb11—C13—H13B	154.2
Sn2—Sn9—Rb12^ii^	64.54 (3)	H13A—C13—H13B	108.0
Sn6—Sn9—Rb12^ii^	122.78 (4)	O10—C14—C19	110.9 (8)
Sn5—Sn9—Rb12^ii^	64.75 (3)	O10—C14—Rb11	47.0 (4)
Sn8—Sn9—Sn7	42.47 (2)	C19—C14—Rb11	79.2 (5)
Sn2—Sn9—Sn7	76.71 (3)	O10—C14—H14A	109.5
Sn6—Sn9—Sn7	42.15 (3)	C19—C14—H14A	109.5
Sn5—Sn9—Sn7	77.70 (3)	Rb11—C14—H14A	89.4
Rb12^ii^—Sn9—Sn7	134.33 (3)	O10—C14—H14B	109.5
O5—Rb10—O7	112.5 (2)	C19—C14—H14B	109.5
O5—Rb10—O12	114.23 (19)	Rb11—C14—H14B	155.5
O7—Rb10—O12	111.1 (2)	H14A—C14—H14B	108.0
O5—Rb10—O6	56.70 (19)	O7—C15—C11	107.9 (8)
O7—Rb10—O6	137.0 (2)	O7—C15—H15A	110.1
O12—Rb10—O6	57.70 (18)	C11—C15—H15A	110.1
O5—Rb10—O4	56.04 (19)	O7—C15—H15B	110.1
O7—Rb10—O4	57.46 (19)	C11—C15—H15B	110.1
O12—Rb10—O4	143.19 (19)	H15A—C15—H15B	108.4
O6—Rb10—O4	104.59 (18)	O5—C16—C28	107.6 (8)
O5—Rb10—O2	142.60 (18)	O5—C16—H16A	110.2
O7—Rb10—O2	56.43 (18)	C28—C16—H16A	110.2
O12—Rb10—O2	55.43 (19)	O5—C16—H16B	110.2
O6—Rb10—O2	104.61 (17)	C28—C16—H16B	110.2
O4—Rb10—O2	108.20 (17)	H16A—C16—H16B	108.5
O5—Rb10—C3	41.0 (2)	O12—C17—C24	109.0 (8)
O7—Rb10—C3	77.8 (2)	O12—C17—H17A	109.9
O12—Rb10—C3	151.7 (2)	C24—C17—H17A	109.9
O6—Rb10—C3	96.9 (2)	O12—C17—H17B	109.9
O4—Rb10—C3	22.9 (2)	C24—C17—H17B	109.9
O2—Rb10—C3	131.0 (2)	H17A—C17—H17B	108.3
O5—Rb10—C24	76.5 (2)	O9—C18—C13	109.9 (8)
O7—Rb10—C24	144.0 (2)	C13—C18—Rb11	77.8 (5)
O12—Rb10—C24	40.6 (2)	O9—C18—H18A	109.7
O6—Rb10—C24	22.1 (2)	C13—C18—H18A	109.7
O4—Rb10—C24	126.6 (2)	Rb11—C18—H18A	95.5
O2—Rb10—C24	94.5 (2)	O9—C18—H18B	109.7
C3—Rb10—C24	117.5 (3)	C13—C18—H18B	109.7
O5—Rb10—C22	150.3 (2)	Rb11—C18—H18B	150.1
O7—Rb10—C22	76.7 (2)	H18A—C18—H18B	108.2
O12—Rb10—C22	40.0 (2)	O3—C19—C14	110.0 (8)
O6—Rb10—C22	96.7 (2)	O3—C19—Rb11	45.2 (4)
O4—Rb10—C22	130.8 (2)	C14—C19—Rb11	77.5 (5)
O2—Rb10—C22	22.7 (2)	O3—C19—H19A	109.7
C3—Rb10—C22	153.5 (3)	C14—C19—H19A	109.7
C24—Rb10—C22	80.5 (2)	Rb11—C19—H19A	93.1
O5—Rb10—C26	77.3 (2)	O3—C19—H19B	109.7
O7—Rb10—C26	40.1 (2)	C14—C19—H19B	109.7
O12—Rb10—C26	147.1 (2)	Rb11—C19—H19B	152.7
O6—Rb10—C26	126.9 (2)	H19A—C19—H19B	108.2
O4—Rb10—C26	22.6 (2)	N1—C20—C10	114.0 (9)
O2—Rb10—C26	96.0 (2)	N1—C20—Rb11	49.5 (5)
C3—Rb10—C26	38.4 (3)	C10—C20—Rb11	75.2 (6)
C24—Rb10—C26	148.7 (2)	N1—C20—H20A	108.8
C22—Rb10—C26	116.8 (3)	C10—C20—H20A	108.8
O5—Rb10—C11	147.5 (2)	Rb11—C20—H20A	154.0
O7—Rb10—C11	39.5 (2)	N1—C20—H20B	108.8
O12—Rb10—C11	76.1 (2)	C10—C20—H20B	108.8
O6—Rb10—C11	126.5 (2)	Rb11—C20—H20B	94.6
O4—Rb10—C11	96.5 (2)	H20A—C20—H20B	107.7
O2—Rb10—C11	22.1 (2)	N2—C21—C2	114.8 (8)
C3—Rb10—C11	117.4 (3)	N2—C21—H21A	108.6
C24—Rb10—C11	116.2 (2)	C2—C21—H21A	108.6
C22—Rb10—C11	37.8 (2)	N2—C21—H21B	108.6
C26—Rb10—C11	79.3 (2)	C2—C21—H21B	108.6
O5—Rb10—C28	39.6 (2)	H21A—C21—H21B	107.5
O7—Rb10—C28	143.5 (2)	O2—C22—C30	108.8 (8)
O12—Rb10—C28	77.1 (2)	O2—C22—Rb10	57.3 (4)
O6—Rb10—C28	21.3 (2)	C30—C22—Rb10	86.1 (6)
O4—Rb10—C28	94.0 (2)	O2—C22—H22A	109.9
O2—Rb10—C28	125.8 (2)	C30—C22—H22A	109.9
C3—Rb10—C28	80.6 (3)	Rb10—C22—H22A	162.9
C24—Rb10—C28	37.2 (2)	O2—C22—H22B	109.9
C22—Rb10—C28	116.9 (2)	C30—C22—H22B	109.9
C26—Rb10—C28	116.3 (2)	Rb10—C22—H22B	70.0
C11—Rb10—C28	147.4 (2)	H22A—C22—H22B	108.3
O9—Rb11—O3	98.3 (2)	O9—C23—C25	109.3 (8)
O9—Rb11—O10	121.43 (19)	O9—C23—Rb11	45.0 (4)
O3—Rb11—O10	59.79 (18)	C25—C23—Rb11	81.0 (5)
O9—Rb11—O1	59.0 (2)	O9—C23—H23A	109.8
O3—Rb11—O1	136.72 (18)	C25—C23—H23A	109.8
O10—Rb11—O1	98.80 (19)	Rb11—C23—H23A	154.5
O9—Rb11—O8	96.40 (19)	O9—C23—H23B	109.8
O3—Rb11—O8	98.59 (18)	C25—C23—H23B	109.8
O10—Rb11—O8	137.29 (18)	Rb11—C23—H23B	88.3
O1—Rb11—O8	118.80 (19)	H23A—C23—H23B	108.3
O9—Rb11—O11	135.5 (2)	O6—C24—C17	110.5 (9)
O3—Rb11—O11	120.77 (18)	O6—C24—Rb10	52.8 (4)
O10—Rb11—O11	97.86 (19)	C17—C24—Rb10	84.9 (6)
O1—Rb11—O11	97.78 (19)	O6—C24—H24A	109.6
O8—Rb11—O11	59.82 (19)	C17—C24—H24A	109.6
O9—Rb11—N1	119.5 (2)	Rb10—C24—H24A	76.5
O3—Rb11—N1	119.6 (2)	O6—C24—H24B	109.6
O10—Rb11—N1	60.5 (2)	C17—C24—H24B	109.6
O1—Rb11—N1	61.2 (2)	Rb10—C24—H24B	161.3
O8—Rb11—N1	119.54 (19)	H24A—C24—H24B	108.1
O11—Rb11—N1	60.6 (2)	N2—C25—C23	113.3 (8)
O9—Rb11—N2	60.7 (2)	N2—C25—H25A	108.9
O3—Rb11—N2	60.6 (2)	C23—C25—H25A	108.9
O10—Rb11—N2	119.8 (2)	N2—C25—H25B	108.9
O1—Rb11—N2	118.9 (2)	C23—C25—H25B	108.9
O8—Rb11—N2	60.08 (19)	H25A—C25—H25B	107.7
O11—Rb11—N2	119.1 (2)	O4—C26—C7	110.6 (8)
N1—Rb11—N2	179.6 (2)	O4—C26—Rb10	55.2 (4)
O9—Rb11—C10	76.9 (2)	C7—C26—Rb10	84.8 (5)
O3—Rb11—C10	129.2 (2)	O4—C26—H26A	109.5
O10—Rb11—C10	79.5 (2)	C7—C26—H26A	109.5
O1—Rb11—C10	21.1 (2)	Rb10—C26—H26A	163.0
O8—Rb11—C10	132.2 (2)	O4—C26—H26B	109.5
O11—Rb11—C10	91.9 (2)	C7—C26—H26B	109.5
N1—Rb11—C10	42.6 (2)	Rb10—C26—H26B	74.1
N2—Rb11—C10	137.6 (2)	H26A—C26—H26B	108.1
O9—Rb11—C14	122.5 (2)	N1—C27—C12	112.3 (9)
O3—Rb11—C14	40.2 (2)	N1—C27—H27A	109.2
O10—Rb11—C14	21.1 (2)	C12—C27—H27A	109.2
O1—Rb11—C14	118.2 (2)	N1—C27—H27B	109.2
O8—Rb11—C14	121.7 (2)	C12—C27—H27B	109.2
O11—Rb11—C14	101.8 (2)	H27A—C27—H27B	107.9
N1—Rb11—C14	79.4 (2)	O6—C28—C16	110.7 (8)
N2—Rb11—C14	100.8 (2)	O6—C28—Rb10	50.2 (4)
C10—Rb11—C14	100.1 (2)	C16—C28—Rb10	84.0 (5)
O9—Rb11—C8	134.1 (2)	O6—C28—H28A	109.5
O3—Rb11—C8	77.9 (2)	C16—C28—H28A	109.5
O10—Rb11—C8	21.2 (2)	Rb10—C28—H28A	159.5
O1—Rb11—C8	92.6 (2)	O6—C28—H28B	109.5
O8—Rb11—C8	129.4 (2)	C16—C28—H28B	109.5
O11—Rb11—C8	78.7 (2)	Rb10—C28—H28B	80.3
N1—Rb11—C8	41.8 (2)	H28A—C28—H28B	108.1
N2—Rb11—C8	138.4 (2)	N2—C29—C5	115.9 (8)
C10—Rb11—C8	71.5 (2)	N2—C29—H29A	108.3
C14—Rb11—C8	37.7 (2)	C5—C29—H29A	108.3
O9—Rb11—C12	127.7 (2)	N2—C29—H29B	108.3
O3—Rb11—C12	134.0 (2)	C5—C29—H29B	108.3
O10—Rb11—C12	92.3 (2)	H29A—C29—H29B	107.4
O1—Rb11—C12	78.7 (2)	O12—C30—C22	108.3 (8)
O8—Rb11—C12	77.2 (2)	O12—C30—H30A	110.0
O11—Rb11—C12	20.9 (2)	C22—C30—H30A	110.0
N1—Rb11—C12	42.4 (2)	O12—C30—H30B	110.0
N2—Rb11—C12	137.2 (2)	C22—C30—H30B	110.0
C10—Rb11—C12	71.1 (2)	H30A—C30—H30B	108.4
C14—Rb11—C12	103.3 (2)	Rb14—Rb13—N4	99.7 (19)
C8—Rb11—C12	71.2 (2)	Rb14—Rb13—Sn5^iii^	87.6 (17)
N3—Rb12—Sn2^i^	99.2 (2)	N4—Rb13—Sn5^iii^	101.4 (2)
N3—Rb12—Sn2^iv^	105.1 (2)	Rb14—Rb13—Sn3^i^	76.9 (14)
Sn2^i^—Rb12—Sn2^iv^	106.32 (4)	N4—Rb13—Sn3^i^	69.2 (2)
N3—Rb12—Sn5^iv^	110.5 (2)	Sn5^iii^—Rb13—Sn3^i^	159.77 (16)
Sn2^i^—Rb12—Sn5^iv^	144.75 (4)	Rb14—Rb13—Sn6^i^	97 (2)
Sn2^iv^—Rb12—Sn5^iv^	48.60 (2)	N4—Rb13—Sn6^i^	104.0 (2)
N3—Rb12—Sn9^iv^	147.0 (2)	Sn5^iii^—Rb13—Sn6^i^	153.00 (12)
Sn2^i^—Rb12—Sn9^iv^	100.78 (4)	Sn3^i^—Rb13—Sn6^i^	44.32 (5)
Sn2^iv^—Rb12—Sn9^iv^	43.83 (2)	Rb14—Rb13—Sn2^i^	122.7 (14)
Sn5^iv^—Rb12—Sn9^iv^	44.22 (2)	N4—Rb13—Sn2^i^	65.8 (2)
N3—Rb12—Rb13	95.0 (2)	Sn5^iii^—Rb13—Sn2^i^	147.86 (8)
Sn2^i^—Rb12—Rb13	59.74 (5)	Sn3^i^—Rb13—Sn2^i^	45.88 (5)
Sn2^iv^—Rb12—Rb13	157.50 (6)	Sn6^i^—Rb13—Sn2^i^	43.71 (5)
Sn5^iv^—Rb12—Rb13	132.44 (6)	Rb14—Rb13—Sn8^iii^	64.7 (13)
Sn9^iv^—Rb12—Rb13	117.68 (6)	N4—Rb13—Sn8^iii^	139.2 (3)
N3—Rb12—Rb12^v^	110.7 (3)	Sn5^iii^—Rb13—Sn8^iii^	43.99 (4)
Sn2^i^—Rb12—Rb12^v^	54.72 (3)	Sn3^i^—Rb13—Sn8^iii^	133.95 (7)
Sn2^iv^—Rb12—Rb12^v^	51.60 (3)	Sn6^i^—Rb13—Sn8^iii^	114.86 (9)
Sn5^iv^—Rb12—Rb12^v^	95.99 (4)	Sn2^i^—Rb13—Sn8^iii^	154.80 (14)
Sn9^iv^—Rb12—Rb12^v^	62.56 (4)	Rb14—Rb13—Rb12	160 (2)
Rb13—Rb12—Rb12^v^	111.99 (6)	N4—Rb13—Rb12	94.5 (2)
N3—Rb12—Rb14	92.5 (4)	Sn5^iii^—Rb13—Rb12	103.20 (8)
Sn2^i^—Rb12—Rb14	59.39 (15)	Sn3^i^—Rb13—Rb12	95.55 (11)
Sn2^iv^—Rb12—Rb14	159.3 (3)	Sn6^i^—Rb13—Rb12	65.89 (9)
Sn5^iv^—Rb12—Rb14	134.4 (3)	Sn2^i^—Rb13—Rb12	51.93 (7)
Sn9^iv^—Rb12—Rb14	120.2 (4)	Sn8^iii^—Rb13—Rb12	111.90 (10)
Rb13—Rb12—Rb14	2.6 (4)	Rb13—Rb14—Sn3^i^	93.4 (12)
Rb12^v^—Rb12—Rb14	112.35 (15)	Rb13—Rb14—Sn5^iii^	83 (2)
C10—O1—C13	112.2 (7)	Sn3^i^—Rb14—Sn5^iii^	166.4 (10)
C10—O1—Rb11	111.6 (5)	Rb13—Rb14—Sn8^iii^	106.4 (15)
C13—O1—Rb11	112.6 (6)	Sn3^i^—Rb14—Sn8^iii^	147.5 (13)
C11—O2—C22	113.1 (7)	Sn5^iii^—Rb14—Sn8^iii^	45.43 (13)
C11—O2—Rb10	102.2 (5)	Rb13—Rb14—Sn6^i^	73.7 (14)
C22—O2—Rb10	100.1 (5)	Sn3^i^—Rb14—Sn6^i^	43.92 (13)
C19—O3—C5	113.4 (7)	Sn5^iii^—Rb14—Sn6^i^	144.5 (8)
C19—O3—Rb11	114.5 (5)	Sn8^iii^—Rb14—Sn6^i^	117.2 (5)
C5—O3—Rb11	114.5 (6)	Rb13—Rb14—Sn4^iii^	127 (2)
C3—O4—C26	113.2 (8)	Sn3^i^—Rb14—Sn4^iii^	136.6 (12)
C3—O4—Rb10	99.4 (6)	Sn5^iii^—Rb14—Sn4^iii^	44.92 (14)
C26—O4—Rb10	102.2 (5)	Sn8^iii^—Rb14—Sn4^iii^	42.5 (2)
C6—O5—C16	112.0 (8)	Sn6^i^—Rb14—Sn4^iii^	150.3 (12)
C6—O5—Rb10	122.0 (6)	Sn3^i^—Rb14—Rb12	87.2 (6)
C16—O5—Rb10	123.2 (5)	Sn5^iii^—Rb14—Rb12	92.3 (9)
C24—O6—C28	111.7 (7)	Sn8^iii^—Rb14—Rb12	104.1 (6)
C24—O6—Rb10	105.1 (5)	Sn6^i^—Rb14—Rb12	58.9 (4)
C28—O6—Rb10	108.5 (5)	Sn4^iii^—Rb14—Rb12	136.2 (8)
C7—O7—C15	112.4 (7)	Sn3^i^—Rb14—Rb10^iii^	103.2 (11)
C7—O7—Rb10	121.3 (6)	Sn5^iii^—Rb14—Rb10^iii^	85.7 (5)
C15—O7—Rb10	122.1 (5)	Sn8^iii^—Rb14—Rb10^iii^	49.7 (5)
C2—O8—C1	112.1 (7)	Sn6^i^—Rb14—Rb10^iii^	103.5 (9)
C2—O8—Rb11	112.0 (5)	Sn4^iii^—Rb14—Rb10^iii^	47.6 (5)
C1—O8—Rb11	112.0 (5)		

Symmetry codes: (i) −*x*+2, −*y*+1, −*z*+1; (ii) *x*, *y*, *z*+1; (iii) −*x*+1, −*y*+1, −*z*+1; (iv) *x*, *y*, *z*−1; (v) −*x*+2, −*y*+1, −*z*.
